# Equity and Pandemic Influenza Vaccine Response: The PIP Framework as a Model for an Efficient Pandemic Influenza Response Through Equitable Vaccine Access

**DOI:** 10.3390/vaccines14080653

**Published:** 2026-07-24

**Authors:** Kate S. Rawlings, Olga Kim, Anne Huvos

**Affiliations:** World Health Emergencies Program, World Health Organization (WHO), 1211 Geneva, Switzerland

**Keywords:** vaccination, pandemic influenza, pandemic preparedness, equity, access and benefit sharing

## Abstract

Equity is fundamental to an effective pandemic vaccination response, yet the COVID-19 pandemic demonstrated how inequitable access, driven by purchasing power and delayed supply for low- and middle-income countries (LMICs), prolonged transmission and enabled viral evolution. The Pandemic Influenza Preparedness (PIP) Framework offers a model for providing timely and equitable access to pandemic influenza vaccines. Through legally binding advance supply agreements, the PIP Framework secures real-time access by the WHO to a proportion of global vaccine production and mandates allocation based on public health risk and need. In doing so, it addresses the two axes of inequity observed in previous pandemics—distribution and timeliness—by ensuring that affected populations that do not have access, irrespective of income status, are not at the back of the queue.

## 1. Introduction

The purpose of a pandemic vaccination program is to reduce morbidity and mortality associated with a pandemic pathogen’s circulation [1]. This is achieved through inducing immunity to the pandemic pathogen within a sufficient proportion of the immunonaïve population through vaccination, limiting the pathogen’s ability to transmit between hosts and thus reducing pathogen circulation [2,3].

To do this efficiently, a pandemic vaccination program must prioritize populations where incidence, prevalence, and transmission risks are highest [1,4,5]. Focusing on these populations decreases the number of immunonaïve hosts in affected areas, thereby limiting transmission and slowing overall spread [4,5].

Lower transmission not only protects individuals from disease but also reduces the likelihood of pathogen mutation [6]. Directing vaccines to affected populations cuts transmission where mutation pressure is highest. This limits opportunities for new variants to emerge, preserving vaccine effectiveness by reducing the risk of immune escape. This benefits all countries, as vaccine effectiveness is critical for reducing the circulation of the pandemic pathogen and thus ending the pandemic [1,6].

Timely vaccination of affected populations, therefore, represents a powerful alignment between equity and efficiency, where the most equitable distribution is also the most efficient means of achieving global pandemic control.

Despite this, equity is rarely the driving force in pandemic vaccination response. This was evidenced in the COVID-19 pandemic, when countries’ purchasing power and vaccine nationalism took precedence. At the beginning of the COVID-19 vaccine response, high-income countries (HICs) received a disproportionate amount of COVID-19 vaccines, allowing them to fully vaccinate 70% of their population within 12 months [7,8]. Due to low and no vaccine availability, low- and middle-income countries (LMICs) were unable to start their vaccination campaigns until months after HICs [9]. After 12 months of vaccine availability, only 4% of people in low-income countries (LICs) were fully vaccinated [8].

Prospective and retrospective modeling of global resource allocation [8,10] indicates that inequitable COVID-19 vaccine distribution prolonged the duration of the pandemic. The inequitable vaccine distribution allowed for mutations to develop due to low vaccination coverage in LMICs [8]. Had vaccines been equitably shared across income groups, infections across LICs, LMICs, and HICs are estimated to have been 25.9%, 12.6%, and 15% lower, respectively, in the first six months of vaccine availability [10]. This reduction would have likely led to a shorter pandemic and fewer global deaths.

Efforts were made for an equitable COVID-19 vaccine response through the COVID-19 Vaccines Global Access (COVAX) Facility; however, due to several challenges, including vaccine nationalism, the COVAX Facility was unable to achieve its targets [11,12].

Although the COVID-19 vaccine response was deeply inequitable, not all pandemic vaccine responses are destined to be so. The COVID-19 pandemic was caused by a novel pathogen, and there were no advance supply agreements or frameworks in place. Had the pandemic been caused by a novel strain of influenza, the vaccine response would have been more equitable due to the Pandemic Influenza Preparedness (PIP) Framework [13]. Developed following the 2009 H1N1 influenza pandemic, and adopted in 2011 by WHO’s 194 Member States, the PIP Framework provides a model of how pandemic vaccination can be delivered equitably, an approach which ultimately benefits everyone.

## 2. The PIP Framework—A Mechanism for an Equitable Pandemic Vaccination Response

The PIP Framework is a unique instrument, specific to pandemic influenza, which takes a holistic approach and provides a model for how pandemic vaccines can be delivered equitably through a global, coordinated pandemic response, allowing for an effective vaccine response. Although developed before the COVID-19 pandemic, the Framework contains provisions which directly address the two axes of inequity observed in the COVID-19 response: delays in timely vaccine access for LMICs and overall access driven by purchasing power. In short, the PIP Framework ensures that LMICs are not at the back of the queue for pandemic influenza vaccines.

The PIP Framework, a WHO Member State negotiated text adopted at the World Health Assembly in May 2011, provides WHO with the mandate to “coordinate influenza pandemic preparedness and response” (p. 15 [13]). It is an example of an Access and Benefit Sharing System, where one of the benefits secured is real-time access to pandemic influenza products developed by manufacturers who have received PIP Biological Material (p. 8 [13]) from the WHO-coordinated Global Influenza Surveillance and Response System (GISRS).

In the 15 years since its adoption, the WHO has managed to secure access to ~11% of the expected global pandemic influenza vaccine production as it is produced. This is through the conclusion of 17 legally binding advance supply agreements (SMTA2s) with manufacturers of pandemic influenza vaccines. These 17 manufacturers are estimated to constitute 87% of future pandemic influenza vaccine production. Based on the latest estimates from the WHO influenza manufacturer survey [14], the WHO would therefore have access to approximately 940 million doses of pandemic influenza vaccine over the first 12-month period of production, in a best-case scenario, through the PIP Framework.

Through securing this access, WHO will be able to ensure that there are pandemic influenza vaccines ready to be provided to affected countries, regardless of income status. Equity is enshrined in the PIP Framework through Section 6.0.2 (iii), which states that vaccines will be prioritized “to developing countries, particularly affected countries, according to public health risk and needs” (p. 15 [13]). The specific criteria for this allocation will be finalized by WHO during the initial stages of the pandemic, as epidemiological factors of the pandemic virus emerge. The Framework thus establishes a process for allocating pandemic influenza vaccines to LICs and LMICs, directly addressing the disproportionate distribution of pandemic vaccines to HICs observed during COVID-19.

Access alone is not sufficient to ensure equity; it is timely access that is critical for reducing virus transmission. Timeliness is addressed by the provisions of the PIP Framework, with advance supply agreements under the PIP Framework specifying that manufacturers will provide WHO with a proportion of their “real-time pandemic vaccine production” (p. 34 [13]). In the instance that a manufacturer has committed 10% of its production to the WHO, for every 100 doses they produce, they must provide 10 to the WHO for donation to countries, based on public health risk and need.

It is the structure of the PIP Framework itself, which contains legally binding commitments for real-time access to pandemic influenza vaccines directly from vaccine manufacturers, that distinguishes it from other equity-driven pandemic instruments, such as the COVAX Facility. PIP does not rely on pooled procurement or coordinated donation efforts to gain access to vaccines. Rather, pandemic influenza vaccines will be available to countries based on public health risk and need, regardless of income status, as soon as manufacturers start producing them. Furthermore, the volume of vaccines available under the Framework will be based on overall global vaccine production at the time of the pandemic, as commitments are based on percentages of production capacity and not static numbers of doses.

## 3. Challenges to Delivering Equitable Vaccine Access Through the PIP Framework

Although equity is at the core of the PIP Framework, there are several external challenges which may hinder the WHO’s ability to deliver pandemic influenza vaccines based on “public health risk and need”(p. 15 [13]). The Framework ensures that the WHO will be able to provide some pandemic influenza vaccine to affected countries without access. This, however, is only an early step in the pandemic vaccine response, which requires that a receiving country be able to accept, license, and administer the vaccine to a population that is willing to accept it, in order to successfully reduce transmission. These aspects were identified as barriers to timely pandemic vaccine roll-out and population uptake in both the 2009 H1N1 and COVID-19 pandemic [12,15,16,17] and would affect LMICs most.

The WHO conducts a wide range of activities to improve these factors of country readiness for pandemic response, a number of which are undertaken using PIP Framework Partnership Contribution (PC) funds—the other key benefit-sharing mechanism of the PIP Framework, which secures annual payments from influenza product manufacturers. WHO uses the funds to strengthen pandemic influenza preparedness capacities at global, regional, and country levels. To date, over US$ 350 million has been collected from manufacturers to support pandemic influenza preparedness activities in 86 countries.

Many of these activities specifically target identified barriers to pandemic vaccination and vaccine uptake, including developing national pandemic response plans and national vaccine deployment plans; working with national regulatory authorities to improve regulatory readiness; and developing local risk communication and community engagement (RCCE) plans and infodemic management systems. Over the 15 years of PIP Framework implementation, there has been significant gains in these areas with PC Funds directly supporting 37 WHO Member States update their pandemic influenza preparedness plans, 16 WHO Member States reach maturity level 3 in the WHO’s classification of regulatory authorities for medicines (meaning it is deemed a stable, well-functioning, and integrated regulatory agency), and 11 WHO Member States develop or update their national deployment and vaccination plans.

Although targeted to pandemic influenza, the preparedness activities have already generated broad collateral benefits to pandemic preparedness and response. For instance, during the COVID-19 pandemic, 47 of the 48 countries receiving regulatory capacity strengthening support under the PIP Framework licensed at least one COVID-19 vaccine. Eight of these countries had not received vaccines during the H1N1 pandemic and were able to license a COVID-19 vaccine within 15 days of the WHO Emergency Use Listing [18]. Despite the efforts to reduce the challenges to equitable pandemic influenza vaccination through the PIP Framework, one key challenge will remain: vaccine hoarding and vaccine nationalism by HICs. This was witnessed during the COVID-19 pandemic and implemented through export restrictions by vaccine-producing HICs [12]. The legally binding advance supply agreements under the PIP Framework, signed between WHO and vaccine manufacturers, are subject to national customs and export regulations. Should certain countries pursue similar vaccine nationalism policies, the WHO’s ability to access the vaccines committed under the PIP Framework would be limited, and the equity provision of the PIP Framework would be significantly undermined.

## 4. Conclusions

Equity is at the core of a successful pandemic vaccine response, with inequitable access ultimately being detrimental to all countries. Timely access to pandemic vaccines for affected populations that do not have such access is a critical component of equity, as it reduces transmission and reduces the risk of pathogen mutation. The PIP Framework provides a model for how timely pandemic influenza vaccine access can be provided to affected countries based on public health risk and need. The PIP Framework also supports preparedness strengthening to ensure that countries provided with vaccines are ready to license and deploy the products. Impediments to the successful roll-out of equity include vaccine nationalism as observed in the COVID-19 pandemic, which would challenge the PIP Framework’s timely access to vaccines. The PIP Framework was negotiated and adopted by WHO’s 194 Member States in the interest of global equity and solidarity, and it is critical that world leaders honor their commitment to equity, as enshrined in the third principle of the PIP Framework, when considering vaccine nationalist policies. The recent adoption of the WHO Pandemic Agreement in May 2025 and entry into force of the amendments to the International Health Regulations (2005) in September 2025 provide a measure of hope that countries are intent on not repeating the inequities seen during the COVID-19 pandemic vaccine response.

## Data Availability

No new data were created or analyzed in this study. Data sharing is not applicable to this article.

## Abbreviations

The following abbreviations are used in this manuscript:

COVAX	COVID-19 Vaccines Global Access
GISRS	Global Influenza Surveillance and Response System
HIC	High-Income Country
LIC	Low-Income Country
LMICs	Low- and Middle-Income Countries
PIP	Pandemic Influenza Preparedness
RCCE	Risk Communication and Community Engagement
SMTA2	Standard Material Transfer Agreement 2
WHO	World Health Organization

## References

[1] Madhav N., Oppenheim B., Gallivan M., Mulembakani P., Rubin E., Wolfe N., Jamison D.T., Gelband H., Horton S., Jha P., Laxminarayan R., Mock C.N., Nugent R. (2017). Pandemics: Risks, Impacts, and Mitigation. Disease Control Priorities: Improving Health and Reducing Poverty.

[2] Fine P., Eames K., Heymann D.L. (2011). “Herd Immunity”: A Rough Guide. Clin. Infect. Dis..

[3] Orenstein W.A., Offit P.A., Edwards K.M., Plotkin S.A. (2023). Plotkin’s Vaccines.

[4] Cao W., Zhu J., Wang X., Tong X., Tian Y., Dai H., Ma Z. (2022). Optimizing Spatio-Temporal Allocation of the COVID-19 Vaccine Under Different Epidemiological Landscapes. Front. Public Health.

[5] Singer B.J., Thompson R.N., Bonsall M.B. (2022). Evaluating Strategies for Spatial Allocation of Vaccines Based on Risk and Centrality. J. R. Soc. Interface.

[6] Cobey S., Larremore D.B., Grad Y.H., Lipsitch M. (2021). Concerns about SARS-CoV-2 Evolution Should Not Hold Back Efforts to Expand Vaccination. Nat. Rev. Immunol..

[7] Mathieu E., Ritchie H., Rodés-Guirao L., Appel C., Gavrilov D., Giattino C., Hasell J., Macdonald B., Dattani S., Beltekian D. (2020). Coronavirus (COVID-19) Vaccinations. Our World Data.

[8] Ye Y., Zhang Q., Wei X., Cao Z., Yuan H.-Y., Zeng D.D. (2022). Equitable Access to COVID-19 Vaccines Makes a Life-Saving Difference to All Countries. Nat. Hum. Behav..

[9] Gozzi N., Chinazzi M., Dean N.E., Longini I.M., Halloran M.E., Perra N., Vespignani A. (2023). Estimating the Impact of COVID-19 Vaccine Inequities: A Modeling Study. Nat. Commun..

[10] Moore S., Hill E.M., Dyson L., Tildesley M.J., Keeling M.J. (2022). Retrospectively Modeling the Effects of Increased Global Vaccine Sharing on the COVID-19 Pandemic. Nat. Med..

[11] World Health Organization Achieving 70% COVID-19 Immunization Coverage by Mid-2022. https://www.who.int/news/item/23-12-2021-achieving-70-covid-19-immunization-coverage-by-mid-2022.

[12] Pushkaran A., Chattu V.K., Narayanan P. (2023). A Critical Analysis of COVAX Alliance and Corresponding Global Health Governance and Policy Issues: A Scoping Review. BMJ Glob. Health.

[13] World Health Organization (2021). Pandemic Influenza Preparedness (PIP) Framework for the Sharing of Influenza Viruses and Access to Vaccines and Other Benefits.

[14] Taaffe J., Goldin S., Lambach P., Sparrow E. (2025). Global Production Capacity of Seasonal and Pandemic Influenza Vaccines in 2023. Vaccine.

[15] Baylor N.W., Goodman J.L. (2022). Vaccine Preparedness for the Next Influenza Pandemic: A Regulatory Perspective. Vaccines.

[16] Fisher D., Hui D.S., Gao Z., Lee C., Oh M., Cao B., Hien T.T., Patlovich K., Farrar J. (2011). Pandemic Response Lessons from Influenza H1N1 2009 in Asia. Respirology.

[17] Hessel L., The European Vaccine Manufacturers (EVM) Influenza Working Group (2009). Pandemic Influenza Vaccines: Meeting the Supply, Distribution and Deployment Challenges. Influenza Other Respir. Viruses.

[18] World Health Organization (2023). Pandemic Influenza Preparedness Framework: Partnership Contribution High-Level Implementation Plan III 2024–2030.

